# Multimodal treatment for liver abscess caused by fish bone migration: a case report

**DOI:** 10.1093/jscr/rjag505

**Published:** 2026-06-25

**Authors:** Akari Sonoda, Kana Ohmoto, Akihiko Togashi, Hideaki Naoe, Tetsuya Okino, Chitoshi Ohara

**Affiliations:** Department of Gastroenterological Surgery, NHO Kumamoto Saishun Medical Center, Kumamoto, Japan; Department of Gastroenterology, NHO Kumamoto Saishun Medical Center, Kumamoto, Japan; Department of Gastroenterological Surgery, NHO Kumamoto Saishun Medical Center, Kumamoto, Japan; Department of Gastroenterology, NHO Kumamoto Saishun Medical Center, Kumamoto, Japan; Department of Gastroenterological Surgery, NHO Kumamoto Saishun Medical Center, Kumamoto, Japan; Department of Gastroenterological Surgery, NHO Kumamoto Saishun Medical Center, Kumamoto, Japan

**Keywords:** fish bone, liver abscess, foreign body migration, percutaneous drainage, laparoscopic surgery

## Abstract

A 77-year-old man with diabetes developed a fever the day after eating black porgy and was admitted to a nearby hospital. Computed tomography revealed an ~3-cm fish bone that had migrated from the duodenum into the liver, with a surrounding liver abscess. His symptoms did not improve with antibiotic treatment, and he was referred to our hospital on Day 3. Gastroduodenoscopy did not identify the fish bone within the duodenal lumen. Although the antibiotic regimen was changed, his fever persisted and his condition worsened. On hospital Day 7, percutaneous transhepatic abscess drainage was performed, after which the inflammatory response and his condition gradually improved. Laparoscopic removal of the fish bone was performed on hospital day 15. The fish bone was identified within the liver, adjacent to the duodenal bulb, and was removed smoothly. The patient’s postoperative course was uneventful.

## Introduction

Liver abscesses caused by foreign bodies are rare [1], and the objects involved may include fish bones, chicken bones, and toothpicks [2]. These abscesses can develop some time after ingestion of a foreign object, which makes diagnosis difficult. If they progress, they can lead to septic shock and multiple organ failure [3]; therefore, careful investigation and accurate diagnosis are important. Basic treatment for liver abscesses caused by foreign bodies includes antibiotic therapy and removal of the foreign body. There is no established consensus on the appropriate timing for surgery. Here, we report a case in which a liver abscess with severe inflammation due to fish bone migration was treated using a multimodal approach, including antibiotic therapy, percutaneous drainage, and laparoscopic removal.

## Case report

A 77-year-old man with a history of diabetes developed a fever the day after eating black porgy and was admitted to a nearby hospital. Initial laboratory tests showed an elevated white blood cell (WBC) count (11 900/μl) and C-reactive protein (CRP) level (23.4 mg/dl). Computed tomography (CT) revealed a linear object ~3 cm in length connecting the duodenum to the liver, with a liver abscess surrounding it (Fig. 1). Based on the patient’s history of ingestion, it was determined that a fish bone had migrated from the duodenum into the liver, resulting in a liver abscess. The patient was hospitalized, and antibiotic therapy (sulbactam/ampicillin) was initiated; however, the fever persisted without improvement. The patient was therefore transferred to our hospital on Day 3.

**Figure 1:**
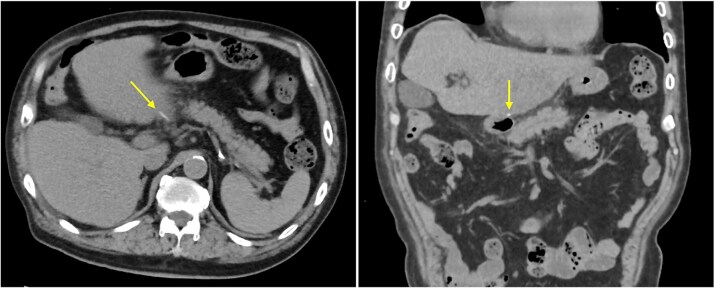
CT image at the previous hospital. A linear high-density object, presumed to be a fish bone, is observed extending from the duodenum to the liver (arrow).

Laboratory tests at our hospital showed a further increase in inflammatory markers (WBC: 16600/μl; CRP: 32.8 mg/dl). CT demonstrated that the fish bone had penetrated further into the liver, with a surrounding liver abscess (Fig. 2). Gastroduodenoscopy did not reveal a fish bone in the duodenum, but a trace of perforation was observed on the anterior wall of the duodenal bulb (Fig. 3). Ultrasonography did not clearly demonstrate the extent of the abscess cavity (Fig. 4a), so the antibiotic regimen was changed to doripenem and conservative treatment was continued. Blood cultures were negative.

**Figure 2:**
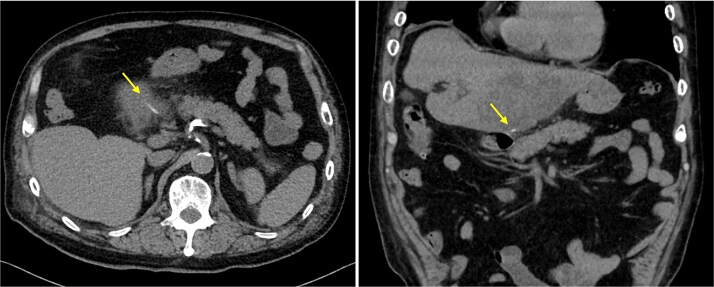
CT image at our hospital. The fish bone is located further within the liver (arrow), with a surrounding liver abscess.

**Figure 3:**
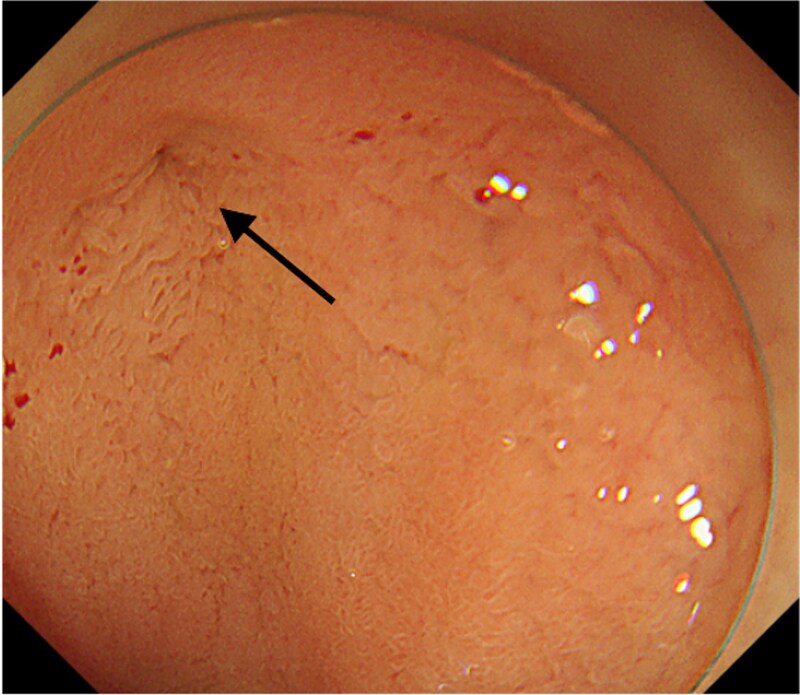
Gastroduodenoscopy image. A trace of perforation is observed on the anterior wall of the duodenal bulb (arrow).

**Figure 4:**
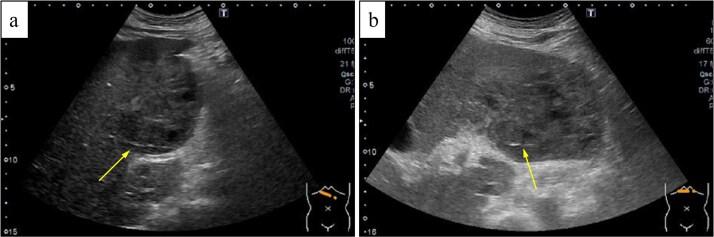
Ultrasonography image. (a) Image at admission to our hospital shows a linear fish bone and a 8-cm mosaic-like abscess surrounding it. (b) Image on hospital day 6 shows the fish bone with an enlarged abscess measuring 11 cm.

The fever persisted and the abscess enlarged (Fig. 4b), so percutaneous transhepatic abscess drainage was performed on Day 7 of hospitalization. A large amount of pus-like fluid was obtained, after which the inflammatory response and the patient’s general condition gradually improved. *Streptococcus anginosus* was detected in the abscess culture.

On Day 15 of hospitalization, laparoscopic surgery was performed to remove the fish bone. No abscess was found in the abdominal cavity, and the fish bone was not immediately identified. After dissection of the area adjacent to the liver near the duodenal bulb, the fish bone was located within the liver and was removed smoothly by traction (Fig. 5). The patient’s postoperative course was uneventful, and he was discharged on postoperative Day 6.

**Figure 5:**
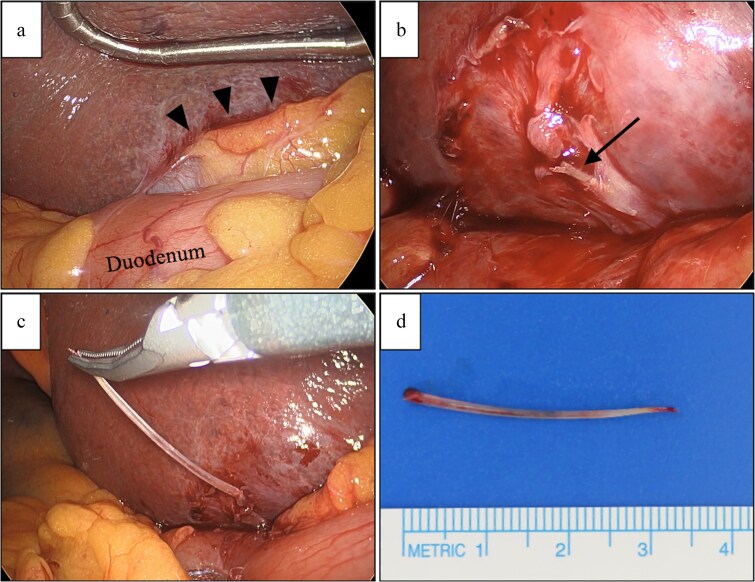
Operative findings and specimen. (a) No abscesses were observed in the abdominal cavity. Dissection was performed between the liver and duodenum (arrowheads). (b) The fish bone was identified (arrow). (c) The fish bone was removed smoothly by traction. (d) The extracted fish bone measured 3.5 cm.

## Discussion

The sites of fish bone migration are commonly the digestive tract and liver [4], and pyogenic liver abscesses secondary to migration are more often observed in the left lobe [5]. Infection control is easier when the fish bone can be removed endoscopically at an early stage [6]. Sim and Sheth [7] reviewed the clinical course of 55 cases of liver abscess caused by fish bones. Six cases resolved without subsequent surgery to remove the foreign body; three were treated with antibiotics alone, and three were treated with antibiotics and percutaneous abscess drainage. A small number of cases were discharged with the fish bone retained; however, there are also reports of recurrent liver abscesses until surgical removal of the foreign body was performed [8].

In the present case, the patient received antibiotic treatment for 3 days at the previous hospital, but the fever persisted and the infection was not controlled. We changed the antibiotic and continued conservative treatment, but the patient’s general condition worsened. Since a relatively high mortality rate (17.6%) has been reported in cases of septic shock due to liver abscess caused by a fish bone [9], percutaneous transhepatic abscess drainage was performed to control the infection. The drainage was effective in this case. Subsequently, surgery was performed on the date requested by the patient, and the patient had a good postoperative outcome. However, if the fish bone had been removed earlier upon admission to our hospital, the abscess might not have progressed and could potentially have been treated more quickly without drainage. Therefore, early surgical removal should be considered when conservative treatment is ineffective.

We performed the surgical removal laparoscopically. Laparoscopic observation revealed no intraperitoneal inflammation, consistent with other reported cases [10]. Preoperative CT was used to estimate the location of the fish bone within the liver. The fish bone was removed smoothly, suggesting that laparoscopic surgery is a useful approach. In addition, preoperative percutaneous drainage may have helped prevent rupture of the abscess into the abdominal cavity.

*Streptococcus anginosus (S. anginosus)*, detected in the abscess culture in this case, is a commensal bacterium of the human mucosa. It is a Gram-positive coccus found in the upper respiratory, gastrointestinal, and urogenital tracts [11]. *Klebsiella* and *Escherichia coli* are more commonly associated with liver abscesses related to biliary tract infections and other causes [12, 13]. In this case, it is likely that *S. anginosus* from the gastrointestinal tract was introduced into the liver via the migrating fish bone, leading to abscess formation. Although the isolated *S. anginosus* was susceptible to the administered antibiotics, antibiotic therapy alone was insufficient to resolve the abscess.

In conclusion, liver abscesses caused by fish bone migration may be difficult to treat with antibiotics alone. Percutaneous drainage can be effective for infection control. Determining the timing of fish bone removal is crucial, and based on the clinical course in this case, early surgical removal appears to be preferable.

## Data Availability

The data reported in this article are unavailable to other researchers because of the need to maintain patient privacy.
